# Clinical Society of Maryland

**Published:** 1893-03

**Authors:** W. T. Watson

**Affiliations:** Secretary; 1519 N. Broadway; Baltimore


### *SOCIETY REPORTS.*

### CLINICAL SOCIETY OF MARYLAND.

BALTIMORE, January 3, 1893.

The Society was called to order by the President, DR. WM. E. MOSELEY.

DR. G. J. PRESTON exhibited a patient suffering from a peculiar nervous affection of his left arm. The patient, a man of twenty-six, has done hard work all his life. He used his left hand rather more than his right. Last October he had a fall and struck on the shoulder and arm of the affected side and the pathological condition developed soon after. The arm is very muscular and there is no evidence of paralysis, no loss of sensation and electric reflexes perfectly normal. The patient's hand assumes all sorts of queer positions due to contracture of the muscles and no sooner is one position reduced than another one immediately ensues, due to the contracture of a different group of muscles. These peculiar phenomena disappear entirely during sleep. Dr. Preston was inclined to believe that the condition was one of hysteria.

DR. WALTER B. PLATT read a paper upon "The Factor of Age in Surgical Operations." His conclusions were based upon operations done by himself upon children and aged people. He has never seen any ill effect in children from confinement to bed for weeks and months in hygienic surroundings. They stand confinement to bed and house better than any other age. In them ether anæsthesia is quickly induced and consciousness quickly regained. He has never seen any bad effects on the circulatory system during or after the administration of anæsthesia in children. The only case of shock which he had seen was in a case of osteo-myelitis of the femur where a great deal of suppuration had been going on for two or three years. A proportionate hemorrhage is probably better borne by a child than by an adult. Children do not tolerate well an operation where the intestines are long exposed. Old persons, having simply the ordinary senile changes but being fairly healthy otherwise, can stand operation very well. Confinement to bed is an important matter in old people. If the injury compels them to lie flat on the back hypostatic pneumonia may put an end to life. They should be watched carefully and propped up if necessary.

DR. RANDOLPH WINSLOW reported "Some Cases of Compound Fracture Occurring in Young Boys."

DR. J. W. CHAMBERS said that children stand the shock moderately well He had recently operated upon a child in its second week for double harelip with protrusion of the premaxillary.

The child was under the anæsthetic for three-fourths of an hour but there was little or no shock. Children stand loss of blood fully as well as other people ; they make blood very rapidly. It is very difficult to say how old people stand shock, for the reason that a large number of the old people operated upon are in a pathological condition, independent of the condition for which they are operated upon. The tissues of old people heal about as well as those of young persons. If the patient's condition warrants an operation and the heart and lungs and other organs are good, the question of age is hardly a factor.

DR. J. D. FARRAR read a paper upon "Intestinal Antisepsis in Typhoid Fever by Means of Bismuth Subiodide and Salol." He commended the plan in practice at the Cooper Hospital, Camden, N. J. He reported a series of twenty-four cases with no deaths. The bismuth and salol were given in five grain doses, alternately, every three hours, night and day. No toxic symptoms from the drugs were noticed. This method of treatment is begun whenever diarrhœa exists and is continued throughout the disease. This treatment is thought to modify the severity if it does not limit the duration of the disease. Tympanites is reduced, diarrhœa controlled and hemorrhage prevented.

Dr. NORMENT said that one of the charts exhibited by Dr. Farrer showed the patient to have had a practically normal pulse, temperature and respiration upon admission, and he thought that the case did not demonstrate so much the benefit of salol and subiodide of bismuth as it did the benefit of convalescence. He thought that further trial was necessary before the utility of the method could be accepted.

W. T. WATSON, Secretary.

1519 *N. Broadway.*

---

BALTIMORE, January 20, 1893.

The Society was called to order by the President, DR. WILLIAM E. MOSELEY.

DR. HARRY FRIEDENWALD exhibited a case of exophthalmos due to idiophatic hemorrhage into the orbit. One morning the patient, a robust negro man, arose feeling quite well. After washing he felt a sudden pain in his eye and found the eye protruding and sensitive. The eyeball was not at all injected and the only reason for the exophthalmos that could be assigned was an orbital hemorrhage. The movements of the eyeball were very much restricted. During the next two days the pain was gradually diminished and the movements were increased. The vision of the affected eye has been greatly impaired since the hemorrhage. No blood had yet appeared under the conjunctiva. There are a great number of cases reported of traumatic orbital hemorrhage with the same symptoms but there are only a few cases of idiopathic hemorrhage on record. The patient's history throws no light upon the cause of the hemorrhage.

DR. A. FRIEDENWALD remembered a similar case which he had seen some years ago. The protrusion of the eyeball came on very suddenly and could be ascribed to no other cause than orbital hemorrhage. These hemorrhages are by no means innocent for the pressure which they exert upon the nerve of the eye is very prejudicial and explains the rapid decrease in the patient's sight. In traumatic cases an accumulation of blood in the orbit is usually followed by total atrophy of the optic nerve.

DR. S. T. EARLE exhibited a Langdon rectal tube. This tube was introduced by Dr. Langdon of the Miami Medical College of Cincinnati, Ohio. It presents many advantages over the tube ordinarily in use. It is five feet in length, being intended to reach from the anus to the cæcum. It is found extremely valuable in rectal alimentation, as the enemata can be carried up all the way into the large bowel and is not followed by a desire for speedy evacuation as is the case when the nourishment is injected only into the rectum. The enemata by this means come into contact with a larger absorbing surface. Another advantage of this tube is its resistance. It is ½-inch tube with only a ⅛-inch opening. This prevents the kinking and bending upon itself which is so likely to take place in the ordinary rectal tube. The opening is at the end and not upon the side. This allows of an easy introduction as by letting the enemata flow in gradually the bowel is distended in advance of the tube. In cases of fecal impaction this resistance and direct opening are of very decided advantage. This tube can be made to pass the fecal obstructions and throw the enemata above the obstruction. It is also valuable in the treatment of oxyuris vermicularis. It has been shown by Heller that the habitat of this worm is about the cæcum and not about the rectum as has been supposed. By this tube our medicaments can be thrown where they will do the most good.

DR. ROBERT W. JOHNSON related a dozen accident cases treated by the blood-clot method. Three gun-shot wounds, two compound fractures of the skull, two compound fractures of patella, one compound fracture of tibia, one compound dislocation of elbow, one punctured wound of knee-joint, one plastic amputation of breast, one strangulated testicle. These cases were all eminently successful. They did not represent all of the series of cases treated by this method but only the most successful ones. While the method is not universally successful, still it may often be trusted, and when it fails it does not leave the patient in a much worse position than had it not been tried.

DR. W. S. HALSTED congratulated Dr. Johnson upon his cases and thought they were excellent illustrations of what could be done by the method.

There is a point not generally understood when the blood-clot method is spoken of. Shede, who advocated this method, made use of it to fill up dead spaces in bone to take the place of sponge, decalcified bone, cat-gut and other foreign materials, and he contradicted the views of Bergmann, who thought that it was absolutely fatal to good results to allow blood to remain in the wounds. He did not, however, ascribe any beneficial antiseptic effects to the blood-clot. He did not know at the time of the experiments of Nuttall, and of others since Nuttall, which showed that the blood of some animals has the power to destroy germs, such as the typhoid germs, when inoculated with these germs. No one has yet succeeded in killing pyogenic organisms with human blood.

Perhaps the value of the method lies in the fact that we are no longer afraid of blood in a wound, and so we do not feel anxious to obliterate dead spaces which are difficult to obliterate and we do not have to prevent very minute arteries from bleeding. We have learned that blood is not very harmful in a wound but we have not learned that it is a thing to be desired in a wound. It is best to have a wound as dry as possible and to close it immediately, provided we can have it dry without constricting tissues with our ligatures and sutures. We have become sure that one of the most important things in technique is to avoid the strangulation of tissue. This is more important than the employment of antiseptics, more important than disinfecting the skin of the patient and perhaps disinfecting our own hands, but not more important than disinfecting the ligatures.

DR. J. E. MICHAEL said that similar cases to those related by Dr. Johnson had been treated successfully by other methods. There can be no object in having a blood-clot except where there is a dead space to deal with. Where wounds can be made clean and closely approximated without constricting tissue it seems reasonable to so treat them rather than to introduce an element which may be harmful.

DR. J. W. CHAMBERS : We have about come to this conclusion, that a blood-clot is probably less injurious to tissue than a drainage tube ; that it does very little harm, whether it does good or not.

DR. RANDOLPH WINSLOW believed that where there were dead spaces, such as cavities in the bone, which heal with difficulty by the process of granulation and cicatrization, the blood-clot affords a scaffolding by which the process of repairs are very much facilitated. He feels safer in making use of drainage where there are considerable cavities which are liable to be filled with fluids which may decompose, but in small cavities or in bone cavities it is frequently desirable to have them filled with blood.

DR. A. FRIEDENWALD : A blood-clot always forms after the enucleation of an eye. Before the period of antisepsis he always expected copious suppuration to follow enucleation and it never failed. With antisepsis he never has suppuration.

DR. JOHNSON said that while the blood-clot will not destroy germs, and he never intended to give the impression that it would, it will allow us to close up our wounds without too much interference. It is nature's method of filling up dead spaces, and is what occurs in subcutaneous wounds and simple fractures. It enables us to do away with the drainage tube to a great extent and this is an extremely valuable addition to surgery. It makes each operation simpler. In endeavoring to check the hemorrhage of small oozing by sponge or gauze or other means we do injury to the tissues. We accomplish a good work by the avoidance of this injury.

W. T. WATSON, Secretary.

1519 *N. Broadway.*

